# Microglia Polarization with M1/M2 Phenotype Changes in rd1 Mouse Model of Retinal Degeneration

**DOI:** 10.3389/fnana.2017.00077

**Published:** 2017-09-05

**Authors:** Tian Zhou, Zijing Huang, Xiaowei Sun, Xiaowei Zhu, Lingli Zhou, Mei Li, Bing Cheng, Xialin Liu, Chang He

**Affiliations:** State Key Laboratory of Ophthalmology, Zhongshan Ophthalmic Center, Sun Yat-sen University Guangzhou, China

**Keywords:** microglia, activation, polarization, neuroinflammation, retinal degeneration, rd1

## Abstract

Microglia activation is recognized as the hallmark of neuroinflammation. However, the activation profile and phenotype changes of microglia during the process of retinal degeneration are poorly understood. This study aimed to elucidate the time-spatial pattern of microglia distribution and characterize the polarized phenotype of activated microglia during retinal neuroinflammation and degeneration in rd1 (*Pde6β*^rd1/rd1^) mice, the classic model of inherited retinal degeneration. Retinae of rd1 mice at different postnatal days (P7, P14, P21, P28, P56, and P180) were prepared for further analysis. We found most CD11b^+^ or IBA1^+^ microglia expressed Ki-67 and CD68 in rd1 mice and these cells migrated toward the layer of degenerative photoreceptors at the rapid rods degeneration phase from P14 to P28. These microglia exhibited typical ameboid activated shape with round bodies and scarce dendrites, while at late phase at P180, they displayed resting ramified morphology with elongated dendrites. Flow cytometry revealed that the percentage of CD86^+^CD206^-^ M1 microglia increased markedly in rd1 retinae, however, no significant change was observed in CD206^+^CD86^-^ M2 microglia. Interestingly, CD86^+^CD206^+^ microglia, an intermediate state between the two extremes of M1 and M2, increased markedly at the rapid rods degeneration phase. The immunofluorescence images revealed that microglia in rd1 mice highly expressed M1 markers including CD16/32, CD86, and CD40. In addition, increased expression of pro-inflammatory cytokines (TNF-α, IL-6, and CCL2) was observed in rd1 mice. Our findings unfolded a panorama for the first time that microglia conducted distinctive behaviors with the progression of retinal degeneration in rd1 mice. Microglia is activated and particularly polarized to a pro-inflammatory M1 phenotype at the rapid rods degenerative phase, suggesting that the involvement of M1 microglia in the retinal neuroinflammation and degeneration. Most microglia adopted an intermediate polarization “M1½” state in rd1, revealing that microglia orchestrated a complicated continuous spectrum in degenerative retina.

## Introduction

Retinitis pigmentosa (RP) is one of the main causes of severe blindness worldwide in 20–64 year olds, particularly in the young. It is characterized by the progressive death of photoreceptors, leading to visual impairment (Wright et al., 2010). Although novel therapies have been advanced greatly in recent years, it is still incurable at present. Multitude of mechanisms have been explored, such as genetic mutations, autophagy deficiency, and neuroinflammation. Sustained inflammation could contribute to the pathological loss of photoreceptor cells in RP and autophagy is implicated as a protective mechanism to resolve neuroinflammation (Cooper et al., 2013; Leinonen et al., 2017). Modulation of retinal inflammatory reaction might be a potential intervention for retinal degeneration (Yoshida et al., 2013a,b).

Microglia, the resident immune cells and primary defenses in retina, promptly react to injury as specialized scavengers. They continuously palpate and monitor the local microenvironment in retina (Hu et al., 2015). When encountering various pathologic insults, such as infection, ischemia and degeneration, microglia become activated promptly. The activation of microglia is recognized as the hallmark of neuroinflammation with typical morphology changes and expression of surface markers (Hendrickx et al., 2013; Heneka et al., 2014). However, the activation profile and phenotype changes of microglia during the process of RP still remain elusive, which are of significance to elucidate the mechanism of retinal neuroinflammation and degeneration.

Previous studies have reported the discovery of microglia activation in the eyes of animal models of RP, such as rd10 (Zhao et al., 2015) and RCS rat (Noailles et al., 2016; Di Pierdomenico et al., 2017), as well as in the patients with RP (Yoshida et al., 2013a). Moreover, several studies have found certain anti-inflammatory drugs that could inhibit microglia activation and alleviate retinal degeneration. However, the efficacy of these drugs was limited and transient, possibly due to the general inhibition of microglia at different disease stages (Bian et al., 2016; Koso et al., 2016; He et al., 2017). In fact, numerous activation statues exist in microglia and microglia with distinctive phenotypes exhibit different roles (Hu et al., 2015).

Microglia in brain have been shown to be highly plastic and could adopt distinctive phenotypes including the classically activated (M1) state and the alternatively activated (M2) state in response to various stimulations (Ma et al., 2016). The M1-like phenotype is characterized by the production of pro-inflammatory mediators including IL-1β, TNF-α, and IL-6 as well as an increased expression of surface markers such as CD16/32, CD86, CD40 and inducible nitric oxide synthase (iNOS), which fuel the inflammatory process (Kalkman and Feuerbach, 2016). Alternatively, microglia could assume an M2 phenotype, which could improve the phagocytosis function and release numerous protective and trophic factors, triggering anti-inflammatory and immunosuppressive responses (Park et al., 2016). For instance, CD206, the classic M2 state marker, is a C-type lectin functions in endocytosis and phagocytosis, and plays an important role in immune homeostasis by scavenging unwanted mannose glycoproteins. Given the similarity of microglia in the retina and brain, we propose that retinal microglia also have the potential to adopt different phenotypes, which may contribute to neuroinflammation during retinal degenerative diseases.

In this study, we investigated the microglia activation and polarization profile in rd1 mice, the classic inherited retinal degeneration model (Farber et al., 1994). Rd1 mouse model presents as an acute autosomal recessive forms of RP. It carries a mutation affecting the expression of β-subunit of PDE coding by *PDE6β* gene, which leads to rod photoreceptor degeneration at postnatal day 8 (P8) and progresses to complete loss of the rods by postnatal week 3, resulting in an early onset severe retinal degeneration (Acosta et al., 2005). Up to now, the microglia phenotypes during retinal degeneration in rd1 mice remain unclear. We aimed to explain the time-spatial pattern of microglia distribution and elucidate the microglia activation and polarization phenotypes in these rd1 mice.

## Materials and Methods

### Animals

Rd1 (FVB/N) mice, an inbred strain with a nonsense mutation in the *PDE6β* gene, were purchased from Beijing Vital River Laboratory Animal Technology, Co., Beijing, China. C57BL/6J mice were purchased from Guangzhou University of Chinese Medicine, Guangzhou, China. Littermates of both sexes ranging from 7 to 180 days old were used in all experiments. Animals were kept in a specific pathogen-free facility and maintained by irradiated sterile diet with clean water. The study protocol was approved by the animal experimental ethics committee of Zhongshan Ophthalmic Center, Sun Yat-sen University, China (authorized number: 2014-039). The methods were carried out in accordance with the approved guidelines of Animal Care and Use Committee of Zhongshan Ophthalmic Center and the Association Research in Vision and Ophthalmology (ARVO) Statement for the Use of Animals in Ophthalmic and Vision Research.

### Immunofluorescence Staining

Eyes were enucleated and fixed in 4% paraformaldehyde (PFA) for 60 min. For cryosection, eyes were embedded in OCT compound (Tissue-Tek; Sakura Fine Technical, Torrance, CA, United States) overnight and 10-μm serial sections were cut through the optic never. For retinal whole mounts, the retinae were dissected out as a cup. Both cryosections and retinal cups were blocked with 0.5% Triton-X100/5% BSA for 2 h at room temperature and incubated with primary antibodies overnight at 4°C. After washing with PBS, the slices were incubated with secondary antibodies for 1 h and counterstained with DAPI at 1:1,000 (Invitrogen) for 5 min at room temperature before mounted.

### Antibodies

The primary antibodies included anti-IBA1 antibody (1:100, Wako), anti-CD68 antibody (1: 100, Abcam, Cambridge, MA, United States), anti-CD16/32 antibody (1:100, BD Biosciences), anti-CD206 antibody (1:100, R&D Systems, Inc., Minneapolis, MN, United States), anti-CD11b antibody (1: 100, Abcam, Cambridge, MA, United States), anti-CD86 antibody (1: 100, Abcam, Cambridge, MA, United States), anti-CD40 antibody (1:100, Abcam, Cambridge, MA, United States), anti-CD163 antibody (1:100, Santa Cruz, MA, United States), anti-TMEM119 antibody (1: 100, Abcam, Cambridge, MA, United States), anti-Rhodopsin antibody (1:100, Santa Cruz, MA, United States). These primary antibodies and abbreviates were explained in Supplementary Table S1, including the description of the characteristics of M1 and M2 markers. Secondary antibodies included donkey anti-rabbit IgG (H+L) Alexa Fluor^®^ 555, goat anti-rat IgG (H+L) Alexa Fluor^®^ 488, donkey anti-rabbit IgG (H+L) Alexa Fluor^®^ 488, and donkey anti-goat IgG (H+L) Alexa Fluor^®^ 555 secondary antibodies (1:800, Invitrogen).

### Image Analysis

Six retinae from six mice were used in each group for retinal whole mounts, and three or four images were randomly captured in each of the center, mid-periphery and periphery area for analysis. As shown in the **Supplementary Figure S1A**, every retina was divided into three regions based on the distance from the optic nerve to the margin of retina (He et al., 2014; Tao et al., 2015). Particularly, the peripheral retina covers the distance from 1500 to 2250 μm since the radius of retina is about 2250 μm. The images were obtained using a Zeiss Axiophot fluorescent microscope and LSCM (LSM510, Carl Zeiss) and processed uniformly using Adobe Photoshop CS5. Quantitative analysis of the microglia in every field/image was carefully delineated based on pixel intensities and was calculated using Image J (National Institutes of Health) and GraphPad Prism (GraphPad Software, Version 6.0, La Jolla, CA, Unites States) (Erny et al., 2015). The dendrites length and the number of dendrites in each microglia were measured with Image-pro plus v6.0 software (Media Cybernetics, Bethesda, MD, United States).

### Flow Cytometry

The eyeballs were enucleated from C57 and rd1 mice at P14, P21, P28, and P180. Retinae were dissected out and placed in 1 ml RPMI with a pH of 7.4 regulated by Hepes (1:100, MP BIOMEDICALS), and disaggregated by gently pipetting up and down through a wide bore pipette tip as previously described (Noailles et al., 2016). This cell suspension was filtered through a 70-μm strainer (BD Biosciences, San Diego, CA, United States) to prevent cell clumps. The cells were washed with phosphate-buffered saline (PBS) containing 1% FBS (Life Technologies, Grand Island, NY, United States) and 1% Hepes (MP BIOMEDICALS), and were stained by CD45 (2 ug/ml, BD pharmingen^TM^), CD11b (5 ug/ml, eBioscience), CD86 (1 ug/ml, BD pharmingen^TM^), and CD206 (5 ug/ml, Biolegend) antibodies for 30 min at room temperature. A four-laser Becton-Dickinson FACS Calibur (BD Biosciences) was used to collect the data, and FlowJo software was used for analysis.

### Real-time PCR

The total RNA of the retinae was extracted with TRIzol (Invitrogen, Carlsbad, CA, United States) and converted into first-strand cDNA using PrimeScript^TM^ RT reagent Kit (TaKaRa Biotechnology, Co., Ltd., Dalian, China) according to the manufacturer’s instructions. Quantitative real-time PCR (qRT-PCR) experiments were performed with SYBR Green I in a Light Cycler 96 (Roche Applied Science, Mannheim, Germany). The primers are listed in Supplementary Table S2. For normalization and relative quantification, the housekeeping GAPDH gene was used as a reference gene. Expression was calculated by relative quantification using the 2^-^^Δ Δ^ ^Ct^ method. Three retinae of three mice were used in each group. A twofold change versus normal controls were considered significant.

### Statistical Analysis

Statistical analysis was performed using GraphPad Prism (GraphPad Software, Version 6.0, La Jolla, CA, United States). For immunofluorescence staining on retinal whole-mount, six retinae from six mice were used in each group, and three or four images were randomly captured in each of the center, mid-periphery and periphery area in every retina for analysis. Representative results are shown in the figures. At least three retinae of three mice were used in experiments including PCR, flow-cytometry, Tunel staining and immunofluorescence on retinal section, which were independently repeated three times. Unpaired Student’s *t*-test was used to compare the means between two groups. An ordinary ANOVA followed by Tukey’s *post hoc* test was performed to compare mean differences in two or more groups. Data are presented as mean ± standard error of the mean (SEM). ^∗^*p* < 0.05 was considered statistically significant.

## Results

### Microglia Presented Distinctive Morphological Change after Activation in Retinal Degeneration of rd1 Mice

**FIGURE 1:**
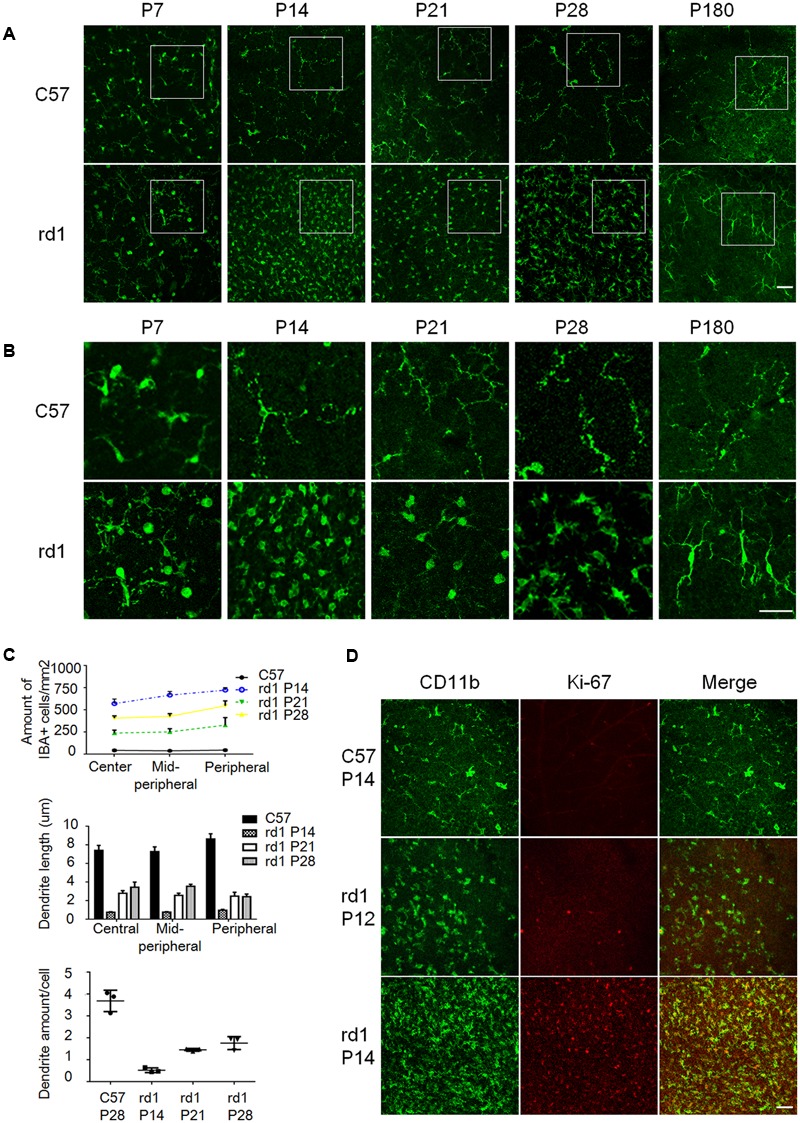
The morphological alterations of microglia during the disease progression. **(A)** The amount of IBA1^+^ microglia increased significantly in rd1 mice from P14 to P28, with the peak at P14. Scale bar = 50 μm. **(B)** Microglia presented typical ameboid shape of activation with round bodies and scarce dendrites during P14 to P28 in rd1 mice. At P14, the dendrite length were the shortest and the amount of dendrites were the least; at P180, elongated but stiff dendrites were observed. Scale bar = 50 μm. **(C)** Graphs showing the calculated microglia amount, dendrite length, and the amount of dendrites of each microglia at different ages (*n* = 6 mice per group, one-way ANOVA and Tukey’s *post hoc* test, *p* = 0.0012, *p* = 0.0113, and *p* = 0.01, respectively). **(D)** Most IBA1 positive microglia expressed Ki-67 at P14 in the ONL, whereas the Ki-67^+^IBA1^+^ cells at P12 were much less than those at P14. Scale bar = 50 μm.

Microglia have been reported to be activated with morphological changes in response to various insults, which contribute to many neurodegenerative diseases (Colonna and Butovsky, 2017; Liddelow et al., 2017). To elucidate the activation status of microglia in rd1 mice of inherited retinal degeneration, we collected retinae from these mice at different ages and analyzed the amount and morphology of microglia using IBA1 immunostaining on retinal whole-mounts to detect the microglia morphology. There was no difference of microglia amount at P7 between rd1 and C57 mice, excluding the presence of impaired development of microglia in rd1 mice. However, more IBA1^+^ microglia were observed in rd1 mice from P14 to P28 with the peak at P14 (**Figures 1A–C**), when the rod photoreceptors experienced cell death dramatically. Of note, the representative images were captured in the peripheral retina, where the microglia accumulated prominently (**Supplementary Figure S1B**). In addition, the microglia in rd1 mice presented typical ameboid activated shape with round bodies and scarce dendrites during P14 to P28, while the elongated dendrites was observed at P180 (**Figure 1B**), indicating that microglia exhibited activated morphology during the rapid photoreceptor degeneration phase and returned to resting statue at the final stage. We also calculated the dendrite length and the amount of dendrites in each microglia and found the amount of microglia was the highest at P14, and the activated microglia at P14 had the shortest length and the least dendrites (**Figure 1C**). The co-staining data of CD11b and Ki-67, a nuclear protein necessary for cellular proliferation, revealed the presence of Ki-67 positive proliferating microglia in rd1 mice but not C57 mice. Of note, these proliferating microglia at P14 increased significantly compared with those at P12, the day with the maximum rods death in rd1 mice (**Figure 1D**).

To further identify the retinal microglia in this study, we also detected CD11b expression and found almost all IBA1 positive cells expressed CD11b (**Supplementary Figure S2A**). In addition, Tmem119, a type IA single-pass transmembrane protein recently reported as specific microglia marker, and CD163, which was exclusively expressed on macrophage and monocyte were also detected. The result showed that most IBA1 positive cells co-stained with Tmem119, but not with CD163 (**Supplementary Figure S2B**), indicating that IBA1 positive cells referred to retinal microglia in this study.

### The Time-Spatial Pattern of Microglia Distribution in rd1 Mice

**FIGURE 2:**
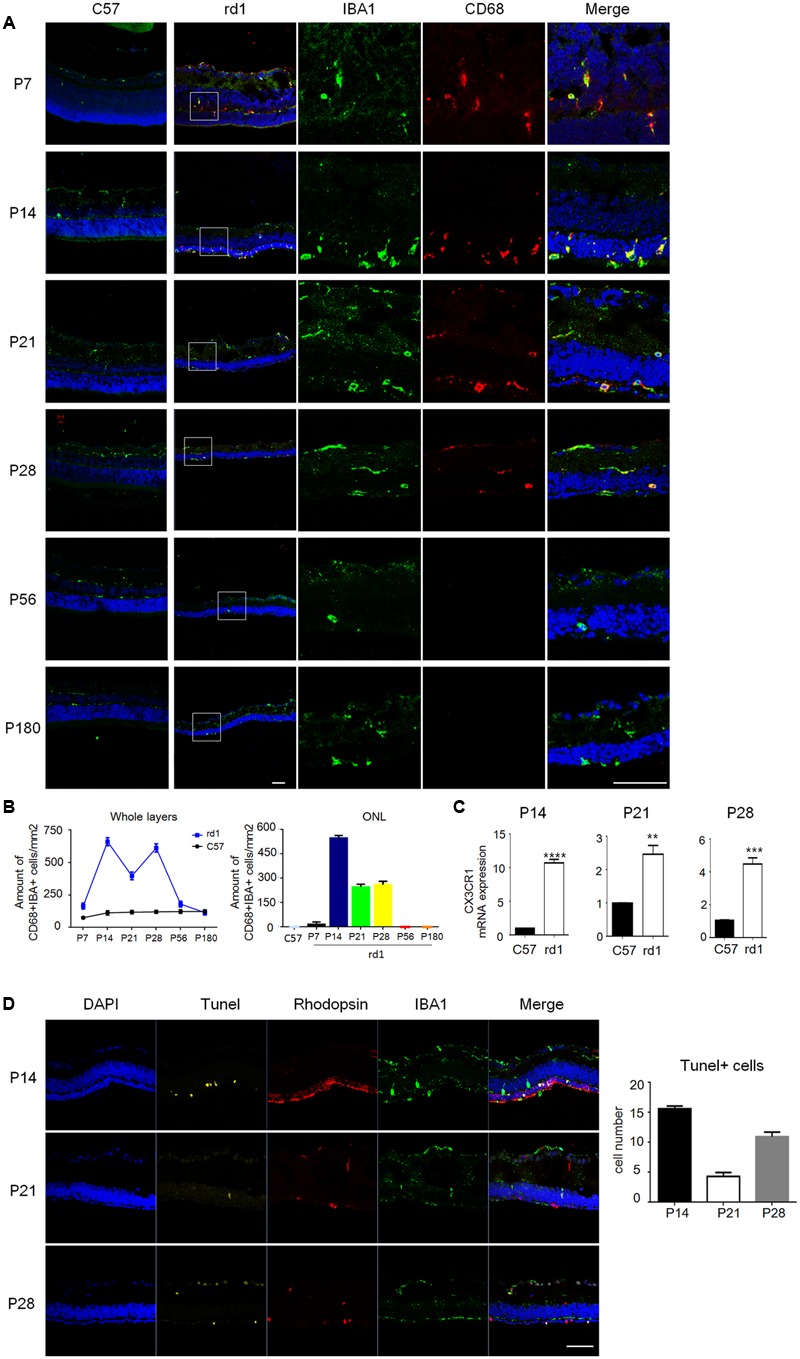
Microglia were activated with distinctive distribution at different disease stages. **(A)** Microglia co-staining IBA1 (green) and CD68 (red) were observed during P7 to P28, while at P56 and P180, CD68 staining was absent. Of note, the total amount of CD68^+^IBA1^+^ microglia increased particularly at P14 and distributed mainly in ONL. Scale bar = 50 μm. **(B)** Graphs showing the calculated CD68^+^IBA1^+^ microglia amount in whole layers and the ONL in retinal sections (*p* = 0.0018, one-way ANOVA and Tukey’s *post hoc* test, *n* = 3 mice per group). **(C)** Real time PCR data showed the expression of CX3CR1 increased significantly in the rd1 retinae at P14 (9.709 ± 0.5237), P21 (1.466 ± 0.2626) and P28 (3.429 ± 0.3770, unpaired Student’s *t*-test, *n* = 3 mice per group. ^∗∗^*p* < 0.01, ^∗∗∗^*p* < 0.001, ^∗∗∗∗^*p* < 0.0001). **(D)** Photoreceptor cells in ONL experienced severe loss with Tunel positive at P14, while the Tunel positive cells appeared not only in the ONL, but also distributed over the entire retina at P28 (*p* < 0.0001, one-way ANOVA and Tukey’s *post hoc* test, *n* = 3 mice per group).

To further elucidate the distribution of microglia, immunostaining was performed on cryosection and the results revealed that the amount and distribution of microglia varied greatly at different disease stages. CD68, a marker of activated microglia, was co-stained with IBA1 during P7 to P28 in rd1 mice, while CD68 staining was absent at P56 and P180 (**Figure 2A**), suggesting microglia were activated at the early disease onset and turned still during later retinal degeneration progress. Consistently, CD68^+^IBA1^+^ microglia elevated to the peak at P14 and accumulated in ONL in rd1 mice, where the photoreceptors degeneration occurred (**Figure 2B**), indicating the close relationship between microglia activation and photoreceptors degeneration. Moreover, we explored the CX3CR1, which mostly expressed on microglia in the retina, and found CX3CR1 also increased significantly at P14, P21, and P28 (**Figure 2C**), indicating that microglia proliferated at the rapid rod degenerative phase in rd1 mice. Interestingly, we found the photoreceptor cells in ONL experienced severe loss with Tunel positive at P14, while the Tunel positive cells appeared not only in the ONL, but also distributed over the entire retina at P28 (**Figure 2D**). Meanwhile, the microglia in rd1 mice proliferated and activated, which is associated with the photoreceptors degeneration with Tunel staining, suggesting the involvement of microglia activation in retinal degeneration. Taken together, these data suggested that microglia presented an activated phenotype and accumulated in the ONL in the rapid rod degenerative phase of rd1 mice.

### Activated Microglia Skew to M1 Polarization during the Retinal Degeneration of rd1 Mice

**FIGURE 3:**
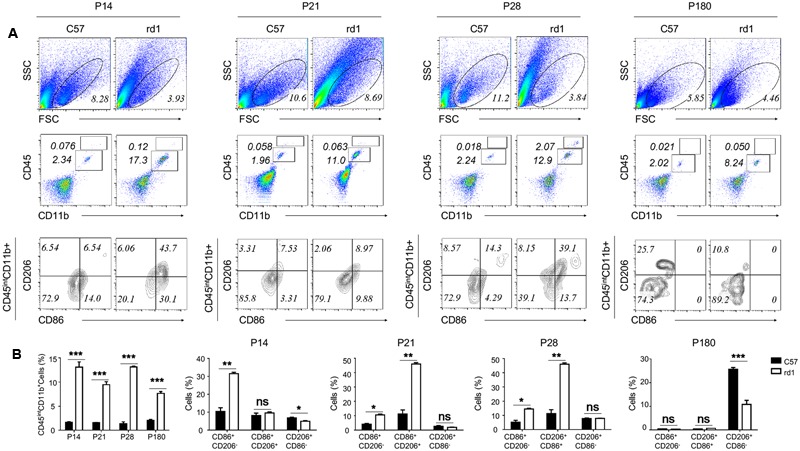
Activated microglia skew to M1 polarization during the retinal degeneration of rd1 mouse. **(A)** Although the gating of alive cells reduced in rd1 retinae comparing with C57 mice, the percentage of microglia increased significantly in rd1 mice. Microglia were identified as CD45^int^CD11b^+^ cells (P14: 11.4767 ± 1.07653, *p* = 0.00043; P21:7.92667 ± 0.615639, *p* = 0.0002; P28: 11.7967 ± 0.27302, *p* < 0.0001; P180: 5.5613 ± 0.3744, *p* = 0.00011). Significant elevation of CD86^+^CD206^-^ population gated on the CD45^int^CD11b^+^ cells was observed in rd1 retinae (P14: 21.1067 ± 2.258, *p* = 0.0007; P21: 6.503 ± 0.9066, *p* = 0.0020; P28: 9.3567 ± 1.4285, *p* = 0.0028), whereas no obvious changes of CD206^+^CD86^-^ population. But at P180, the CD206^+^CD86^-^ M2 microglia was significantly reduced in rd1 mice comparing to C57 mice (14.9 ± 1.13, *p* < 0.0001). Interestingly, the CD86^+^CD206^+^ phenotype markedly increased in microglia from rd1 mice at P21 and P28 (P21: 9.36 ± 1.428, *p* < 0.0003; P28:34.723 ± 2.933, *p* = 0.0003). **(B)** Graph displaying the calculated percentage of CD45^int^CD11b^+^, CD86^+^CD206^-^, CD206^+^CD86^-^, and CD86^+^CD206^+^ cells at each time point (unpaired Student’s *t*-test. *n* = 3 mice per group. ^∗^*p* < 0.05, ^∗∗^*p* < 0.01, ^∗∗∗^*p* < 0.001, ns: no significance).

Microglia could experience M1 and M2 polarization after activation in the brain, involving in the process of neurodegeneration and repair (Nakagawa and Chiba, 2015; Xiong et al., 2016). To study the polarization phenotype of activated microglia in rd1 mice, flow-cytometry was performed on single cell suspension of the whole retina. The results revealed that the total amount of alive retinal cells reduced markedly in rd1 mice compared with same-age control mice due to the degenerative pathology. However, the microglia, presented as CD45^int^CD11b^+^ cells, increased significantly in rd1 mice, corroborated the presence of microglia proliferation. CD86 was recognized as the classical M1 marker and CD206 as the M2 marker. The CD86^+^CD206^-^ M1 microglia were dominant in rd1 mice at different time points while there were no significant changes of CD206^+^CD86^-^ M2 microglia within 28 days. However, the CD206^+^CD86^-^ M2 microglia was significantly reduced in rd1 mice at P180, indicating the impaired M2 microglia in rd1. Interestingly, the phenotype of CD86^+^CD206^+^, a wandering state between the two extremes of M1 and M2, markedly increased at P21 and P28 comparing to the controls (**Figure 3**).

### Profile of Pro-inflammatory M1-Microglia during the Disease Onset

**FIGURE 4:**
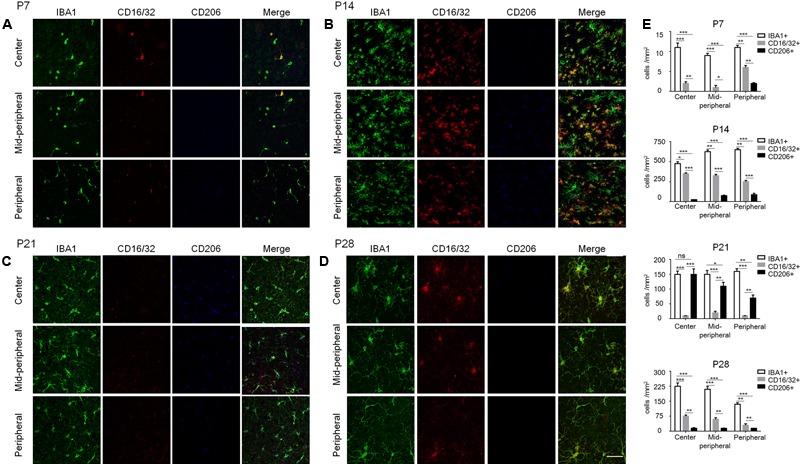
Polarization states of ONL microglia during rd1 retinal degeneration. **(A–D)** Representative co-staining images with CD16/32, CD206, and IBA1 captured at the center, mid-periphery, and periphery retina from rd1 mice at P7, P14, P21, and P28, respectively. **(A)** At P7, CD16/32^+^ microglia appeared in the whole retina, while a few CD206^+^ cells were present in the periphery. **(B)** At P14, the amount of microglia increased dramatically and most of them expressed CD16/32 all over the retinae. **(C)** At P21, CD16/32^+^ microglia decreased significantly while some expressed CD206. **(D)** At P28, the IBA1^+^ microglia as well as CD16/32^+^ and CD206 expression were reduced mildly compared to P21, whereas more IBA1^+^ and CD16/32^+^ cells than those at P7. Scale bar = 50 μm. **(E)** Graph displaying the calculated amount of IBA1^+^, CD16/32^+^, CD206^+^ cells at different ages from P7 to P28 (^∗^*p* < 0.05, ^∗∗^*p* < 0.01, ^∗∗∗^*p* < 0.001, one-way ANOVA and Tukey’s *post hoc* test, *n* = 6 mice per group).

**FIGURE 5:**
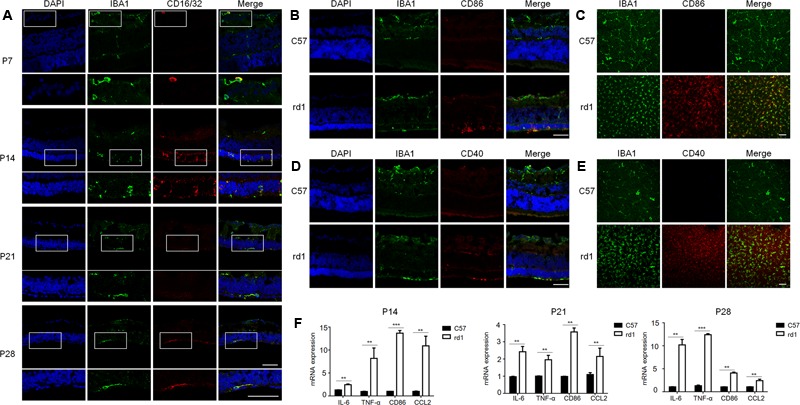
Pattern of pro-inflammatory M1-microglia during the disease onset. **(A)** IBA1^+^CD16/32^+^ microglia were distributed mainly in ONL specifically at P14 when retina experienced the severest loss of photoreceptors. Scale bar = 50 μm. **(B–E)** Microglia in the ONL at P14 in rd1 mice could co-stain with other M1 markers, CD86 and CD40. Scale bar = 50 μm. **(F)** rd1 mice displayed significant increase of M1 related molecules and pro-inflammatory factors at P14, P21, and P28 comparing with C57 mice of the same age, with specifically prominent elevation of CD86 and CCL2 at P14 and significant increase of TNF-α and IL-6 cytokines of over 10-time-fold change at P28 (^∗^*p* < 0.05, ^∗∗^*p* < 0.01, ^∗∗∗^*p* < 0.001, unpaired Student’s *t*-test, *n* = 3 mice per group).

To further understand the microglia polarization states specifically in the degenerative ONL, microglia in the deepest layers were observed by immunostaining on retinal whole mounts and cryosections. We found that the amount of microglia increased significantly and most of them expressed the M1 marker CD16/32 especially at P14. On the other hand, the expression of CD206 (M2 marker) stayed quite low at different time points during the acute degeneration (**Figures 4A–E**). The data of cryosections also revealed that CD16/32^+^IBA1^+^ M1 microglia were distributed mainly in ONL from P14 to P28, particularly at P14 (**Figure 5A**). The expression of CD86 and CD40, the antigen presentation receptors which could fuel the pro-inflammatory responses, were also investigated to access the potential pro-inflammatory profile of M1 microglia. The data showed that both CD86 and CD40 were expressed on IBA1 microglia at P14 (**Figures 5B–E**), suggesting that the microglia were activated and mainly polarized toward to M1 phenotype at the rapid photoreceptor degeneration phase. The M1 related pro-inflammatory factors were examined by real-time PCR. The data showed TNF-α, IL-6, CD86 and the chemokine CCL2 were up-regulated significantly in the rd1 mice at P14, P21, and P28. Interestingly, the prominent elevation of CD86 and CCL2 was observed at P14, while TNF-α and IL-6 displayed more than 10-time-fold increase at P28 (**Figure 5F**).

## Discussion

In this study, we unfolded a panorama for the first time that microglia conducted distinctive behaviors with the progression of retinal degeneration in rd1 mice. During the rapid phase of rod degeneration within 1 month, microglia proliferated and accumulated in the degenerative ONL layer and displayed typical activated morphological changes with ameboid shape. Meanwhile, most of the activated microglia skew to the M1 polarization, presented with higher expressions of M1 markers including CD86, CD16/32 and CD40, as well as pro-inflammatory cytokines and chemokine. The photoreceptor degeneration was associated with microglial M1 polarization in rd1 mice, suggesting the involvement of M1-like pro-inflammatory microglia in the retinal neuroinflammation and degeneration.

Microglia have been reported to be activated and could migrate toward the inflamed site in response to injuries (Xu et al., 2007; Paques et al., 2010). In consistent with previous studies, our data also revealed that in the rd1 mice with *Pde6β* gene mutation, microglia were activated and infiltrated into photoreceptor-located ONL layer where the death of photoreceptors mostly occurred (Wunderlich et al., 2010). Other studies suggested that the close interaction between migrated microglia and mutation-bearing rod photoreceptors was intended to eliminate the degenerative photoreceptors and protect the remaining intact cells, which could have facilitated microglia to phagocyte damaged photoreceptors (Noailles et al., 2016; Roche et al., 2017). However, the migrated microglia induced excessive inflammation through producing large amounts of pro-inflammatory cytokine and promoted the photoreceptors degeneration (Zabel et al., 2016; Lin et al., 2017). In addition, in this study, microglia mainly accumulated in the peripheral rd1 retina where there were more Tunel positive cells (Punzo and Cepko, 2007), corroborated the fact that microglia become activated and migrate to degenerative site, constituting one critical component of neuroinflammation in retinal degeneration. Inflammasome is also of particular importance in the development of inflammatory responses in neurodegenerative diseases (Guo et al., 2015). Aggregated α-synuclein in Parkinson’s disease could trigger IL-1β generation depending on the NLRP3 inflammasome in brain microglia (Saijo et al., 2013; Mohamed et al., 2015; Zhou et al., 2016). Recently, one study reported inflammasome was required in microglial activation and polarization to a pro-inflammatory M1 state *in vitro* (Gaikwad et al., 2017). It is valuable to study on inflammasome in M1 retinal microglia in the future study.

Emerging evidence suggested the ‘activated microglia’ worked as a double-edged sword. Despite most literatures suggested the detrimental effect of microglia activation (Hong et al., 2016; Burma et al., 2017), increasing studies showed the beneficial effect exerted by activated microglia (Colton, 2009; Kigerl et al., 2009). It has been proven that the imbalance of pro- and anti-inflammatory macrophages/microglia played an essential role in neurological disorders and diseases such as ALS and Rett syndrome (Boillee et al., 2006; Wang et al., 2015). Actually, as highly plastic cells, microglia could form a continuum states with distinct phenotypes and functions in the brain (Hughes, 2012; Wang et al., 2013; Nakagawa and Chiba, 2015; Xiong et al., 2016; Lan et al., 2017). Retinal microglia were recognized to share similarities and exhibit some differences with microglia in brain (Ramirez et al., 2017). In this study, we firstly reported the plastic behavior and the profile of microglia with a prominent M1 state and lack of M2 phenotype in rd1 model of RP. The reversion to M1 phenotype contribute to pro-inflammatory environment and has the potential to impair retinal function. The M1 microglia would induce the activation of NADPH oxidase and iNOS and promote generation of proinflammatory cytokines and chemokine (Zeng et al., 2014). Among them, TNF-α and IL-6 have the potential to exacerbate neuronal death and CCL2 is a strong activator of microglia and promotes efficient recruitment and activation of phagocytes to the sites of photoreceptor degeneration (Starossom et al., 2012; Kroner et al., 2014). On the contrary, M2 microglial phenotype was recognized to be neuro-protective in neurodegenerative diseases in brain. It could restore CNS homeostasis in response to short-lasting and low intensity neuronal damage, counteracting neuroinflammation and promoting regeneration (Miron et al., 2013). The absence of M2 phenotype was observed in rd1 mice, suggested the potential M2 role of microglia may be impaired during the rapid rod degeneration phase.

Interestingly, we found many activated microglia characterized by CD86^+^CD206^+^ phenotype, suggesting a wandering state between the two extremes of M1 and M2. Okuno colleagues reported that analysis of the candidate genes in polarized microglial transcriptome failed to differentiate M1 from M2 type polarization signature in ALS transgenic mice (Tada et al., 2014), indicating the presence of non-M1 and non-M2 phenotype. Kumamoto et al. also suggested an intermediate state of polarization (“M1½”) exist and blurred the M1, M2 macrophage line (Knudsen and Lee, 2016; Kumamoto et al., 2016). Indeed, increasing evidence suggested that microglia orchestrated a continuous spectrum but not the simplistic dichotomous M1, M2 distinction of microglia activation. In this study, microglia in rd1 mice displayed a wandering phenotype, which possibly accommodate the fluctuating degeneration rates of rods and cones as well as other retinal cells. The exact role and mechanism would be very important in further investigation.

## Conclusion

Our results showed the panorama of activation profile of microglia in the degenerative retina of rd1 mice. The activated microglia adopted M1-dominent polarization at the rapid rods degenerative process, which is closely associated with pathological changes of retinal degeneration, while there was lack of M2-dominent microglia. An intermediate polarization (“M1½”) state of activated microglia characterized by CD86^+^CD206^+^ phenotype was present, suggesting that microglia orchestrated a complicated continuous spectrum but not the simplistic dichotomous M1, M2 distinction in degenerative retina. Taken together, these findings provided a novel promising therapeutic avenue with re-educating microglia to limit their pro-inflammatory M1 function and enhance M2 phenotype for retinal degenerative diseases.

## Author Contributions

CH conceived the project, and TZ, ML, and BC designed the experiments; TZ, ZH, XS, XZ, and LZ performed the experiments and analyzed the data; TZ and CH wrote the article; CH and XL supervised the project.

## Conflict of Interest Statement

The authors declare that the research was conducted in the absence of any commercial or financial relationships that could be construed as a potential conflict of interest.

## Supplementary Material

FIGURE S1 A schematic presentation of the positions analyzed on a retinal whole mount. **(A)** Schematic drawing showing the center, mid-peripheral and peripheral areas in retina. **(B)** The peripheral area showed in the retinal whole mounts at different time points. Scale bar = 500 μm.

FIGURE S2 Identification of retinal microglia. **(A)** Almost all IBA1 microglia were co-stained with CD11b. **(B)** Most IBA1 positive cells were co-stained with specific microglia marker Tmem119, but not with macrophage marker CD163. Scale bar = 50 μm.
