# Supplementary figures and images for: Microglia Polarization with M1/M2 Phenotype Changes in rd1 Mouse Model of Retinal Degeneration
Source: Front Neuroanat. 2017 Sep 5;11:77. doi: 10.3389/fnana.2017.00077 (PMC5591873; doi:10.3389/fnana.2017.00077)

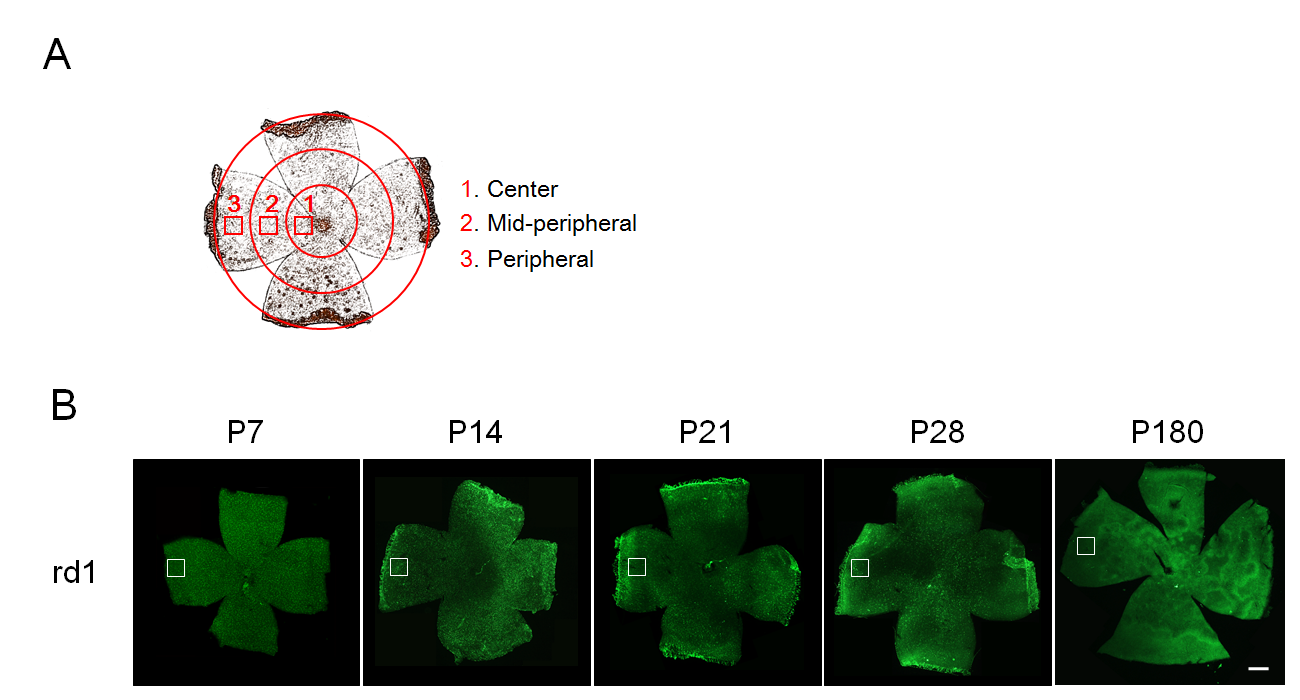

Supplement: FIGURE S1 — A schematic presentation of the positions analyzed on a retinal whole mount. (A) Schematic drawing showing the center, mid-peripheral and peripheral areas in retina. (B) The peripheral area showed in the retinal whole mounts at different time points. Scale bar = 500 μm. [file Image_1.TIF]

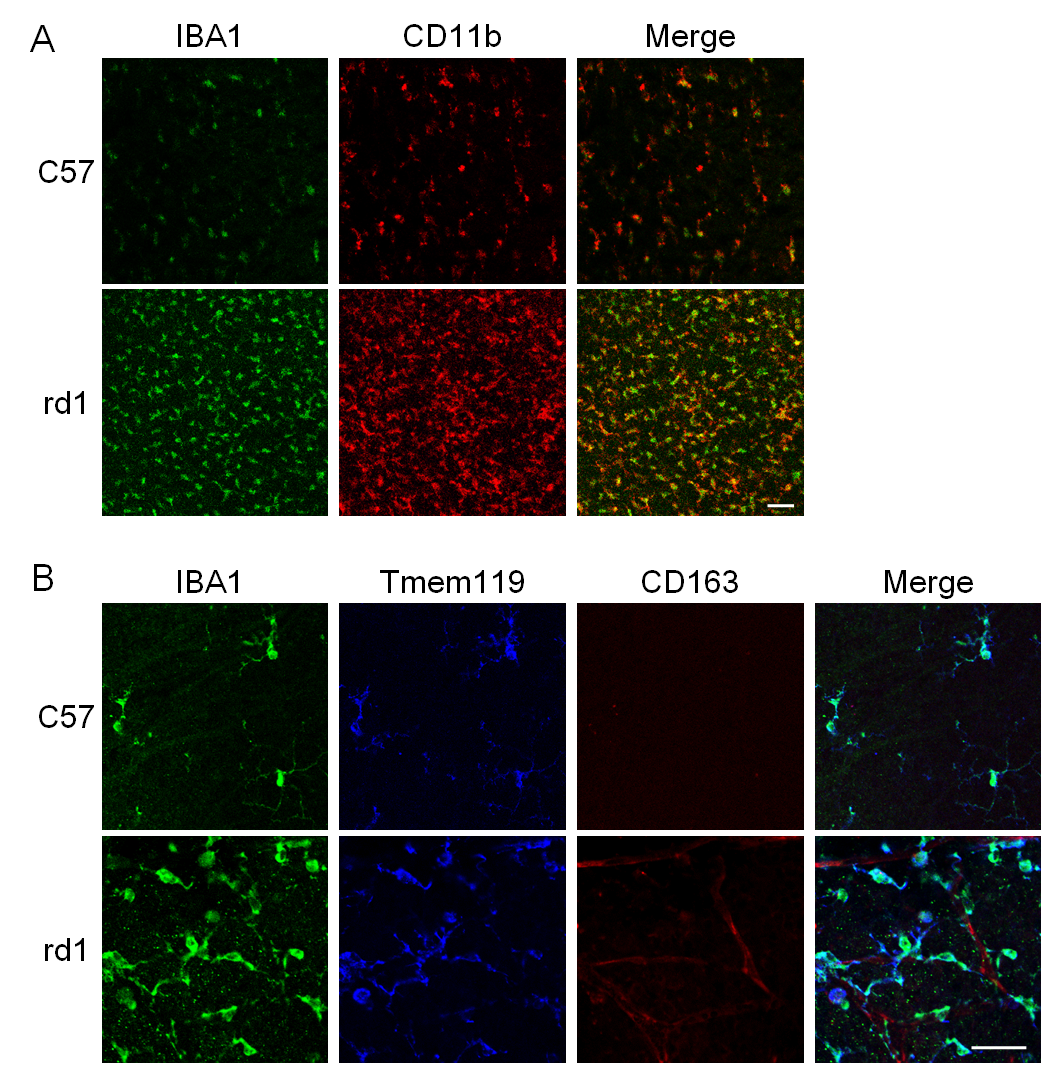

Supplement: FIGURE S2 — Identification of retinal microglia. (A) Almost all IBA1 microglia were co-stained with CD11b. (B) Most IBA1 positive cells were co-stained with specific microglia marker Tmem119, but not with macrophage marker CD163. Scale bar = 50 μm. [file Image_2.TIF]
