# Supplementary material for: Microglia Polarization with M1/M2 Phenotype Changes in rd1 Mouse Model of Retinal Degeneration
Source: Front Neuroanat. 2017 Sep 5;11:77. doi: 10.3389/fnana.2017.00077 (PMC5591873; doi:10.3389/fnana.2017.00077)
Supplement: Supplementary file 3 [file Table_1.docx]

Table S1: Antibodies used in the study

| Abbreviates | Description | Source | Dilution | Host | Application | References |
| --- | --- | --- | --- | --- | --- | --- |
| IBA1 | IBA1^+^ myeloid cells were designated as microglia and/or macrophages | Wako Cat. No. 019-19741 | 1:100 | Rabbit | IF | (Le Blon et al., 2014) |
| CD11b | It’s the β-integrin marker of microglia, as a binding protein for intracellular cell adhesion molecule-1 and complement C3bi. | Abcam ab8878 | 1:100 | Rat | IF | (Roy et al., 2006) |
| CD11b | Microglia possess the phenotype CD45^int^ CD11b^+^ in flow cytometry | eBioscience Percp-Cyaline5.5  LOT: E08304-1634 | 1:100 | Rat | FC | (Locatelli et al., 2012) |
| CD45 |  | BD Pharmingen^TM^  APC LOT: 5174809 | 1:100 | Rat | FC | (Abcouwer et al., 2013) |
| CD68 | Microglia/macrophage activation marker.  Could play a role in phagocytic activities;  Binds to tissue and organ specific lectins or selectins, allowing macrophage/microglia to crawl over other cells | Abcam ab53444 | 1:100 | Rat | IF | (Kobayashi et al., 2013) |
| CD86 | M1 marker. Activated after contact with neutrophils expressing CD28, signaling via NF-κB to induce numerous pro-inflammatory cytokines, such as IL-6 | BD Pharmingen^TM^  PE LOT: 5174609 | 1:100 | Rat | FC | (Louveau et al., 2015) |
|  |  | Abcam ab119857 | 1:100 | Rat | IF |  |
| CD16/32 | M1 marker. FcγRII and FcγRIII for IgG, mediating the release of inflammatory mediators, cytotoxic triggering, and phagocytosis | BD Pharmingen^TM^ LOT: 553141 | 1:100 | Rat | IF | (Higashi et al., 2017) |
| CD40 | M1 marker. CD40 drives retinal protein nitration in vivo and causes NOS2 up-regulation and nitric oxide production | Abcam ab58391 | 1:100 | Rabbit | IF | (Chen et al., 2006). |
| CD206 | M2 marker. A C-type lectin functions in endocytosis and phagocytosis, and plays an important role in immune homeostasis by scavenging unwanted mannoglyco-proteins. | Biolegend FITC Lot:B172941 | 1:100 | Rat | FC | (Azad et al., 2014) |
|  |  | R&D Systems Lot: WFT0115021 | 1:100 | Rat | IF |  |
| CD163 | A marker of perivascular macrophages, with no expression on ramified microglia | Santa Cruz  sc-33560 | 1:100 | Goat | IF | (Prinz et al., 2011) |
| TMEM119 | Microglia marker. A stable and robustly expressed microglial marker for both mouse and human. | Abcam ab209064 | 1:100 | Rabbit | IF | (Bennett et al., 2016) |
| Rhodopsin | A light-sensitive receptor protein involved in visual phototransduction. in the retinal rod photoreceptors | Santa Cruz  sc-57432 | 1：100 | Mouse | IF | (Di Pierdomenico et al., 2017) |

IF: Immunofluorescence; FC: Flow cytometry

References

Abcouwer, S.F., Lin, C.M., Shanmugam, S., Muthusamy, A., Barber, A.J., and Antonetti, D.A. (2013). Minocycline prevents retinal inflammation and vascular permeability following ischemia-reperfusion injury. *J Neuroinflammation* 10**,** 149.

Azad, A.K., Rajaram, M.V., and Schlesinger, L.S. (2014). Exploitation of the Macrophage Mannose Receptor (CD206) in Infectious Disease Diagnostics and Therapeutics. *J Cytol Mol Biol* 1.

Bennett, M.L., Bennett, F.C., Liddelow, S.A., Ajami, B., Zamanian, J.L., Fernhoff, N.B., Mulinyawe, S.B., Bohlen, C.J., Adil, A., Tucker, A., Weissman, I.L., Chang, E.F., Li, G., Grant, G.A., Hayden Gephart, M.G., and Barres, B.A. (2016). New tools for studying microglia in the mouse and human CNS. *Proc Natl Acad Sci U S A* 113**,** E1738-1746.

Chen, K., Huang, J., Gong, W., Zhang, L., Yu, P., and Wang, J.M. (2006). CD40/CD40L dyad in the inflammatory and immune responses in the central nervous system. *Cell Mol Immunol* 3**,** 163-169.

Di Pierdomenico, J., Garcia-Ayuso, D., Pinilla, I., Cuenca, N., Vidal-Sanz, M., Agudo-Barriuso, M., and Villegas-Perez, M.P. (2017). Early Events in Retinal Degeneration Caused by Rhodopsin Mutation or Pigment Epithelium Malfunction: Differences and Similarities. *Front Neuroanat* 11**,** 14.

Higashi, Y., Aratake, T., Shimizu, S., Shimizu, T., Nakamura, K., Tsuda, M., Yawata, T., Ueba, T., and Saito, M. (2017). Influence of extracellular zinc on M1 microglial activation. *Sci Rep* 7**,** 43778.

Kobayashi, K., Imagama, S., Ohgomori, T., Hirano, K., Uchimura, K., Sakamoto, K., Hirakawa, A., Takeuchi, H., Suzumura, A., Ishiguro, N., and Kadomatsu, K. (2013). Minocycline selectively inhibits M1 polarization of microglia. *Cell Death Dis* 4**,** e525.

Le Blon, D., Hoornaert, C., Daans, J., Santermans, E., Hens, N., Goossens, H., Berneman, Z., and Ponsaerts, P. (2014). Distinct spatial distribution of microglia and macrophages following mesenchymal stem cell implantation in mouse brain. *Immunol Cell Biol* 92**,** 650-658.

Locatelli, G., Wortge, S., Buch, T., Ingold, B., Frommer, F., Sobottka, B., Kruger, M., Karram, K., Buhlmann, C., Bechmann, I., Heppner, F.L., Waisman, A., and Becher, B. (2012). Primary oligodendrocyte death does not elicit anti-CNS immunity. *Nat Neurosci* 15**,** 543-550.

Louveau, A., Nerriere-Daguin, V., Vanhove, B., Naveilhan, P., Neunlist, M., Nicot, A., and Boudin, H. (2015). Targeting the CD80/CD86 costimulatory pathway with CTLA4-Ig directs microglia toward a repair phenotype and promotes axonal outgrowth. *Glia* 63**,** 2298-2312.

Prinz, M., Priller, J., Sisodia, S.S., and Ransohoff, R.M. (2011). Heterogeneity of CNS myeloid cells and their roles in neurodegeneration. *Nat Neurosci* 14**,** 1227-1235.

Roy, A., Fung, Y.K., Liu, X., and Pahan, K. (2006). Up-regulation of microglial CD11b expression by nitric oxide. *J Biol Chem* 281**,** 14971-14980.
