# Supplementary material for: Microglia Polarization with M1/M2 Phenotype Changes in rd1 Mouse Model of Retinal Degeneration
Source: Front Neuroanat. 2017 Sep 5;11:77. doi: 10.3389/fnana.2017.00077 (PMC5591873; doi:10.3389/fnana.2017.00077)
Supplement: Supplementary file 4 [file Table_2.docx]

Table S2 Primer list

| Primer name | Forward | Reverse |
| --- | --- | --- |
| TNF-α | GAGGCCAAGCCCTGGTATG | CGGGCCGATTGATCTCAGC |
| CCL2 | CAGCCAGATGCAATCAATGCC | TGGAATCCTGAACCCACTTCT |
| IL-6 | ACTCACCTCTTCAGAACGAATTG | CCATCTTTGGAAGGTTCAGGTTG |
| CD86 | TCAATGGGACTGCATATCTGCC | GCCAAAATACTACCAGCTCACT |
| GAPDH | GCCAAGGCTGTGGGCAAGGT | TCTCCAGGCGGCACGTCAGA |
